# Anti-inflammatory Activity of Tocotrienols in Age-related Pathologies: A SASPected Involvement of Cellular Senescence

**DOI:** 10.1186/s12575-018-0087-4

**Published:** 2018-11-20

**Authors:** Marco Malavolta, Elisa Pierpaoli, Robertina Giacconi, Andrea Basso, Maurizio Cardelli, Francesco Piacenza, Mauro Provinciali

**Affiliations:** Advanced Technology Center for Aging Research, Scientific Technological Area, IRCCS INRCA, via Birarelli 8, 60121 Ancona, Italy

## Abstract

Tocotrienols (T3) have been shown to represent a very important part of the vitamin E family since they have opened new opportunities to prevent or treat a multitude of age-related chronic diseases. The beneficial effects of T3 include the amelioration of lipid profile, the promotion of Nrf2 mediated cytoprotective activity and the suppression of inflammation. All these effects may be the consequence of the ability of T3 to target multiple pathways. We here propose that these effects may be the result of a single target of T3, namely senescent cells. Indeed, T3 may act by a direct suppression of the senescence-associated secretory phenotype (SASP) produced by senescent cells, mediated by inhibition of NF-kB and mTOR, or may potentially remove the origin of the SASP trough senolysis (selective death of senescent cells). Further studies addressed to investigate the impact of T3 on cellular senescence “in vitro” as well as in experimental models of age-related diseases “in vivo” are clearly encouraged.

## Introduction

Tocotrienols (T3) have been shown to represent a very important part of the vitamin E family since they have opened new opportunities to prevent or treat a multitude of age-related chronic diseases [44]. Experiments conducted in both mice and humans have shown potential health benefits from T3 supplementation, including a distinctive and effective anti-inflammatory activity. They were shown to exert a lipid-lowering effect and a superior anti-inflammatory activity compared to tocopherols in cardiovascular diseases (the other class of Vitamin E compounds) [71, 92]. The anti-inflammatory activity of T3 has been also proposed as the main mechanism of action of T3 explaining the amelioration of conditions related to a diet-induced metabolic syndrome in rats [88]. The anti-inflammatory activity of T3 has been also proposed to contribute to their protection against neurodegenerative diseases, including Alzheimer’s disease (AD) [90], and alcohol-induced cognitive impairment in rats [82]. Suppression of inflammation has been also proposed among the mechanisms by which T3 can counteract the ability of cancer cells to proliferate, metastasize, evade apoptotic signals, and develop chemoresistance [54]. Last but not least, low intake and serum levels of tocopherols and T3 have been associated with several age-related pathologies including osteoporosis, sarcopenia and cognitive impairment [72].

In this review, we summarize the broad range of anti-inflammatory effects of T3 on aging and the main age-related diseases with the aim to provide a common mechanistic rationale through which tocotrienols may exert their pro-longevity and pro-health action. In particular, we suggest that part of the anti-inflammatory effects of these natural compounds can be due to their modulation of the senescence-associated secretory phenotype (SASP) produced by senescent cells (hence the meaning of the “SASPected” term in title), which accumulation in aging has been proposed as a key pathological mechanism in different age-related pathologies.

## Influence of Tocotrienols on Lifespan in Model Organisms and on Biomarkers of Aging

Experimental evidence has supported a role of T3 in modulating several mechanisms associated with aging.

The effect of Vitamin E supplementation on lifespan has been analysed in various model organisms of increased complexity, including single-cell organisms and rotifers, nematodes, flies, mice and rats [15]. Most of the studies conducted in single cell organisms, rotifers, and nematodes reported an increase in the mean lifespan without any effect on the maximal lifespan. In almost all these studies, organisms were supplemented with a-tocopherol, some of them with γ-tocopherol, and only one analysed the effect of T3 supplementation.

T3 supplementation was performed in the model-system nematode, *C. elegans.* The treatment reduced the accumulation of protein carbonyl (a good indicator of oxidative damage during aging) and extended of 20% the mean lifespan, but not the maximum lifespan [1]. A tocotrienol-rich fraction (TRF) from palm oil (composed of α-tocopherol, 22%; α-tocotrienol, 24%; γ-tocotrienol, 37%; δ-tocotrienol 12%) also recovered the shortened mean lifespan induced by ultraviolet B irradiation. The administration of 8 or 80 mg/ml of TRF to *C. elegans* resulted in an extension of the mean lifespan, whereas 80 mg/ml of α- tocopherol alone did not. This effect was attributed to the higher antioxidant activity of T3 compared to tocopherols [1]. Importantly, T3 are recognized among those compounds that are able to activate the nuclear factor erythroid-2-related factor 2 (Nrf2) [38], which modulates the transcription of a multitude of cytoprotective genes and is argued to be a lifespan and healthspan extending factor.

Unfortunately, no studies have been conducted until now to evaluate the effect of T3 on the lifespan of insects, rodents, or humans.

However, some studies have provided evidence that treatment with T3 may partly affect some biomarkers of aging. In humans aged above 50 years, TRF supplementation for six months decreased DNA damage [8] and reduced the levels of advanced glycosylation end products (AGE) and protein carbonyls [9], which play important roles in aging, diabetes and cardiovascular diseases. A study investigated whether TRF can modulate collagen synthesis and degradation in human diploid fibroblasts (HDFs) exposed to a dose of H_2_O_2_ able to induce premature senescence. TRF upregulated collagen genes, type I and type III procollagen synthesis, and downregulated matrix metalloproteinases (MMPs) genes and activity in HDFs, suggesting that TRF may protect the skin from aging by enhancing collagen synthesis and inhibiting collagen degradation [37].

Based on this evidence and on the limited toxicity of these natural compounds, it is not surprising that T3 supplementation has become an interesting intervention to target fundamental aspects of aging that play a role in the onset and progression of age-related diseases.

## Tocotrienol Supplements in the Prevention and Treatment of Age-Related Pathologies

Experimental and clinical evidence has been provided that T3 supplements may exert beneficial effects in different age-related pathologies. Moreover, T3 have demonstrated their effectiveness also in chronic inflammatory diseases where tocopherols do not seem to determine similar benefits.

In the subsequent text, we will discuss these distinctive properties of T3 in age-related pathologies.

### Cardiovascular and Metabolic Diseases

T3 have been widely studied for their vascular- and cardio-protective properties. T3 supplement may be able to ameliorate the profile of important risk factors in the development of cardiovascular diseases, such as cholesterol and hypertension. The ability of T3 supplements to reduce cholesterol levels has been demonstrated both in animals [46, 64, 95] and humans [62, 63, 65, 69]. The most impressive reduction of cholesterol was reported in chickens consuming diets supplemented with gamma- and delta-T3. Indeed, these animals showed a reduction of 32% total cholesterol and 66% LDL cholesterol after T3 supplementation [95] which was not obtained with alpha-tocopherol supplementation. Similarly, gamma- and delta-T3 supplements led to a significant decrease in total and low-density lipoprotein (LDL) cholesterol in genetically hypercholesterolemic pigs [64] and humans [67, 68]. Conversely, alpha-tocopherol supplements did not contribute to this effect [45] and may even interfere with T3 activity [69]. Administration of T3 in the form of a tocotrienol-rich fraction (TRF25) yielded a similar reduction of total and LDL-cholesterol with a concomitant decrease of apolipoprotein B and triglycerides compared to the baseline values in hypercholesterolemic humans [69]. The effect of T3 on cholesterol levels was demonstrated to be dependent by their ability to downregulate the 3-hydroxy-3-methyl-glutaryl-coenzyme A reductase (HMG-CoA reductase, HMGR) enzyme which is the rate-limiting enzyme of the cholesterol biosynthetic pathway. The mechanism of their hypolipidemic action seems to involve a posttranscriptional suppression of HMGR [59] by a process that involves ubiquitination and degradation of the enzyme [76]. However, there are also controversial findings to those showing substantial cholesterol reduction after T3 supplementation. For example, a study performed with commercially available tocotrienol supplements at a level of 200 mg total T3/day showed no measurable beneficial effect on key CVD risk factors (including blood cholesterol) in highly compliant adults with elevated blood cholesterol concentrations [49]. Interestingly, a synergistic effect of TRF25 (but not alpha-tocopherol) and lovastatin, a widely used drug (an HMG-CoA reductase inhibitor) to reduce lipid levels in hypercholesterolemic subjects [67, 68] has been also reported, thus suggesting that T3 may also act with different mechanism of actions from HMG-CoA reductase inhibition.

Besides to their effect on lipid metabolism, T3 may influence cardiovascular system in other ways. It has been shown that treatment with T3 can reduce the size of atherosclerotic lesions in Apo-E-deficient mice (−/−) (a mouse model that develop complex atherosclerotic lesions similar to those of humans). The authors observed that the reduction in the size of atherosclerotic lesions was not explained by alterations in lipid metabolism and hypothesized that the effects were the consequence of a reduction of inflammation [67, 68].

Indeed, treatment with T3 is able to suppress the induction of tumor necrosis factor (TNF), Interleukin-1 beta (IL-1β) and interleukin-6 (IL-6) in lipopolysaccharide (LPS) stimulated peritoneal macrophages obtained from 6-week-old BALB/c female mice [66]. Moreover, the effects of T3 on plaque size of Apo-E-deficient mice were retained (even if to a lesser extent) when the treatment was delayed from 6 to 16 weeks, thus suggesting that T3 may act even after atherosclerotic lesions have been developed. Although the authors did not investigate if T3 may affect the accumulation of senescent cells and SASP production, the results are very similar to those obtained with the experimental removal of senescent cells in atherosclerosis-prone low-density lipoprotein receptor-deficient (Ldlr−/−) mice [7]. In this study, it has been shown that senescent macrophages, endothelial cells, and vascular smooth muscle cells drive the atherosclerotic pathology by increasing expression of key atherogenic and inflammatory cytokines and chemokines. Indeed, the clearance of senescent cells from advanced lesions inhibits both plaque growth and maladaptive plaque remodelling processes associated with plaque rupture. The similarity between the two studies may suggest that the beneficial effects on the atherosclerotic model displayed by T3 could be the consequence of their capacity to modulate the production of SASP or the propensity of senescent cells to undergo apoptosis.

A consequence of arterial stiffness is hypertension (high blood pressure). In this process, chronic inflammation produced by senescent cells has been proposed to play a major role [20]. Interestingly, T3 administration determined a significant reduction of blood pressure and improvement in other cardiovascular, metabolic and inflammatory markers in spontaneously hypertensive rats [55], in diet-induced metabolic syndrome in rats [6, 88] as well as in high fat diet-fed rats [87].

#### Cancer

One of the first evidence supporting the protective effect of T3 in cancer was based on a study focused on the role of various high-fat diets in 7,12-dimethylbenz(a) anthracene induced mammary tumorigenesis [78]. In this study, experimental animals supplemented with 20% *w*/w crude palm oil (among vegetable oils, palm oil is the richest natural source of tocotrienols, with 600–1000 ppm concentration corresponding to approximately 0.08% in weight), had a lower incidence of mammary tumors compared to lard fat. Moreover, it was subsequently demonstrated that the protective effect disappears when the palm oil diet is stripped of T3 [53].

Another study showed that supplementation with a TRF from palm oil can delay the onset, incidence, and size of the tumors in nude mice inoculated with MCF-7 breast cancer cells compared to non-supplemented controls [51].

Further studies demonstrated that various T3 isomers exert anticancer effects, which are not generally evident with tocopherol-rich vitamin E preparations [43]. These studies underlined the concept that T3 rather than tocopherols are endowed of potent anticancer effects. In particular, it has been found that T3 (but not α-tocopherol) can suppress the growth of human breast and colorectal cancer cells “in vitro” [14, 52] as well as the responsiveness of human breast cancer cells to chemotherapeutic drugs [19]. In mice, the administration of T3 showed a life-prolonging effect from transplanted tumors, which was not replicated by treatment with tocopherol, [30].

Extensive “in vitro” and “in vivo” evidence has demonstrated that δ- and γ-T3 are the most potent anticancer forms of natural vitamin E [50].

Various antioxidant-independent mechanisms have been proposed to explain the anticancer effect of T3, and, among these, the main are represented by induction of apoptosis, inhibition of cancer cells proliferation, inhibition of angiogenesis, and induction of cellular senescence [38, 50, 86]. In various work focused on the anticancer effect of α-, γ-, and δ-T3 “in vitro”, it has been found that the anticancer action of T3 was related to the induction of mitochondrial dysfunction and apoptosis as well as to the expression of cellular senescence markers [61, 85].

In the HER-2/neu transgenic mouse model (a model which spontaneously develop mammary tumors), dietary supplementation with T3 (90% δ-T3 and 10% γ-T3) delayed the development of tumors and reduced the number and the volume of tumor masses and the size of lung metastases [60]. The beneficial effects of T3 were associated with a reduction of HER-2/neu mRNA and p185HER-2/neu protein and an increase of markers related to cellular senescence in mammary glands while no immune modulation was observed.

T3 have demonstrated their efficacy also in prostate cancer. Both γ- and δ-T3 were shown to inhibit the growth of androgen-dependent and androgen-independent prostate cancer cells with a higher efficacy compared with tocopherols [5]. The antiproliferative effect increased when T3 were administered to prostate cancer cells together with docetaxel [93]. Moreover, a mixed-T3 diet supplemented to a transgenic adenocarcinoma mouse prostate (TRAMP) model induced a lower incidence of tumor formation along with a significant reduction of the levels of high-grade neoplastic lesions as compared to untreated transgenic controls [3]. This reduction was found to be associated with an increased expression of proapoptotic proteins BAD (Bcl2 antagonist of cell death), cleaved caspase-3 and cell cycle regulatory proteins including the cyclin-dependent kinase inhibitors p21 and p27. More recently, using different cell-based assays with non-small-cell lung cancer (NSCLC) models, δ-T3 was found to inhibit cell proliferation, cell migration, invasion, aggregation, and adhesion with a mechanism involving the increase of miR-451 expressions and the downregulation of Notch-1-mediated nuclear factor-κB (NF-κB) [70]. NF-κB is particularly relevant to chronic inflammation and cancer. Many studies showed an anti-tumorigenic and pro-survival role of NF-κB in cancer cells, and recent findings suggest that NF-κB participates in the production of SASP by senescent cells [24] which, in turn, are suspected to promote cancer in old age [84]. Interestingly, the suppression of NF-κB has been reported among the effects of the senolytic drug ABT263 in bone marrow stromal cells from old mice [29], thus suggesting that also T3 may act trough targeting senescent cells.

#### Neurodegenerative Diseases

T3 have been shown to exert some kind of protection in neurodegenerative diseases. In the pathogenesis of major neurodegenerative diseases, oxidative damage may lead to massive neuronal loss via glutamate toxicity. Studies “in vitro” on HT4 hippocampal neuronal cells demonstrate that nanomolar amounts of α-T3, but not α-tocopherol, blocked glutamate-induced death by suppressing glutamate-induced early activation of c-Src kinase [73]. After oral administration, T3 have been shown to cross the blood-brain barrier and to reach brain tissue, thus suggesting that the results “in vitro” can be transferred “in vivo”.

Indeed, chronic treatment with T3 prevented intracerebroventricular streptozotocin-induced cognitive impairment and oxidative-nitrosative stress in rats [81].

These protective effects of T3 have been attributed in large part to their antioxidant effects, thus suggesting that in these circumstances T3 and tocopherols may display similar results. In agreement with this hypothesis, it has been found that AD and MCI patients can display lower serum levels of total tocopherols, total tocotrienols, and total vitamin E compared with cognitively normal subjects [42]. Moreover, elevated levels of tocopherol and T3 forms were associated with reduced risk of cognitive impairment in a Finnish cohort of 140 non-cognitively impaired elderly subjects followed-up for 8 years [41]. However, even if epidemiological studies have evidenced some benefits, the effects of tocopherol and T3 in AD and other neurodegenerative diseases are still under debate. Besides the unambiguous positive effects of T3 in oxidative stress reduction, studies in neuronal cell lines have shown that T3 treatment may also increase amyloid-β (Aβ) levels as well as the activity of enzymes responsible for Aβ production [18]. When T3, in the form of TRF, were tested for 10 months in the AD mouse model APPswe/PS1dE9 (60 mg/kg body weight, daily from 5 to 15 months of age), the treatment mitigated Aβ depositions in the cortex and thioflavin-S-positive fibrillar type plaques in the hippocampus and improved cognitive function [22]. Paradoxically, these results were not associated with a reduction in microglia activation and inflammation, likely due to insufficient dosing, suboptimal method of administration, low bioavailability or late age of initiation. Conversely, in rats postnatally exposed to ethanol, T3 treatment has been shown to counteract all the behavioral, biochemical and molecular alterations observed in different brain regions, including the increase of neuroinflammatory mediators (TNF-α, IL-1β, and TGF-β1) [80]. Unfortunately, there is a lack of information about the effects of T3 on cell senescence markers and Tau protein accumulation, which is the most common pathology among neurodegenerative diseases, including AD. A recent work performed in postmortem human brain tissues and animal models of AD demonstrate that Tau protein aggregation is associated with cellular senescence in the brain [48]. Additional studies addressing the effects of T3 on these pathological markers would be useful to understand if T3 are able to target senescent cells in neurodegenerative diseases.

#### Sarcopenia

Sarcopenia is a disease associated with the aging process and is characterized by loss of muscle mass and strength which in turn affects balance, gait and overall ability to perform tasks of daily living. Both the number and regenerative ability of satellite cells decline during aging through apoptosis-induced cell death or cellular senescence [13, 77]. The decline in the ability of satellite cells to engage in the myogenic process contributes to sarcopenia-induced muscle atrophy.

Some studies focused on the effect of TRF on replicative senescence-associated oxidative stress and on the regenerative capacity of myoblasts in stress-induced premature senescence (SIPS). These studies provide evidence that TRF is able to ameliorate antioxidant defense mechanisms and to improve replicative senescence-associated oxidative stress in myoblasts [28], as well as to recover the normal morphology of SIPS-induced myoblasts [34]. In this last model, TRF treatment also reduced the activity of senescence-associated β-galactosidase (SA-β-gal) and increased cell proliferation [34]. These results suggest that TRF may partly reverse myoblasts senescence and replenish the regenerative capacity of these cells. An alternative interpretation of the results that is based on the hypothesis that T3 may exert senolytic activity has been also recently proposed [40]. Senolytic activity has not been tested for T3 but part of metabolic and apoptotic pathways affected by these compounds in cancer cells overlap with those of quercetin, which has been reported to display this activity in irradiation-induced senescent endothelial cells [96]. This suggests that the rejuvenating effects of T3 on senescent cells might be the net results of a senolytic activity on senescent cells and a selective survival of a sub-population of non-senescent cells in the culture. The promising results “in vitro” suggest that T3 with their antioxidant and anti-inflammatory capabilities may mitigate age-associated skeletal dysfunction and enhance muscle regeneration, thus attenuating sarcopenia. However, “in vivo” studies with preclinical and clinical model will be necessary to verify the potential of T3 for the treatment of this age-related disease.

## Inhibition of Cellular Senescence and Inflammation as Main Mechanisms of Tocotrienol Action in Age-Related Diseases

### Anti-Inflammatory Properties of Tocotrienols

Growing evidence has indicated that T3 exhibit potent anti-inflammatory activity. These effects have been largely attributed to the inhibitory effect of T3 on the proteasome. In fact, besides to be involved in protein degradation for antigen processing, the proteasome represents a central regulator of the inflammation process by controlling the production of inflammatory mediators. Several independent experimental models have suggested that T3 block the activation of nuclear factor kappa-light-chain-enhancer of activated B cells (NF-kB), a master regulator of the inflammatory response. In particular, T3 were found to reduce the activation of NF-kB determined by tumor necrosis factor α (TNF-α), interleukin-1 (IL-1) and phorbol ester. Treatment with T3 has been reported to reduce lipopolysaccharide-induced TNF-α in BALB/c mice, suggesting their potential beneficial anti-inflammatory effects in atherosclerosis [66]. In other studies, α-T3 was shown inhibit in a dose- and time-dependent manner the surface expression of vascular cell adhesion molecule-1 (VCAM1) in TNF-α activated human umbilical vascular endothelial cells (HUVEC) with the subsequent decrease in monocytic cell adherence [56, 79]. In agreement with the majority of studies on the anti-inflammatory activity of T3, these results were mediated by an inhibition of NF-kB binding activity [79]. A decrease of the expression of inflammatory cytokines supported by the inhibition of NF-kB expression was also observed in human monocytic cells treated with palm oil-derived TRF [89]. T3 have been also shown to modulate the activity of the signal transducer and activator of transcription 3 (STAT3), another transcription factor downstream of mTOR, which has been associated with inflammation, proliferation, and tumorigenesis [11]. In particular, it has been shown that γ-T3 inhibits both induced and constitutive activation of STAT3 in cancer cell lines [54].

Another relevant aspect of T3 is their ability to suppress transforming growth factor β (TGF-β) signaling, a cytokine characterized by a pleiotropic role in the inflammatory processes. In fact, while the delivery of TGF-β has proven beneficial in allograft rejection and autoimmunity, TGF-β can also contribute to oxidative stress and DNA damage during induction of cellular senescence [31]. In particular, TGF-β is involved in the induction of p21-dependent cellular senescence during mammalian embryonic development [47] as well as in experimental models of hepatocellular carcinoma [74] and mesenchymal stromal cells [25]. Moreover, aging imposes an elevation of TGF-β signaling in the neurogenic niche of the hippocampus and in the myogenic niche of skeletal muscle [94]. This change induces a switch in the activity of TGF-β, which became a pro-inflammatory factor instead of retaining its canonical role in attenuating immune responses. The suppression of TGF-β signaling with a single drug (an Alk5 Type I receptor kinase inhibitor) was found to simultaneously enhance neurogenesis and muscle regeneration in old mice. Similarly, T3 have been reported to inhibit the activity of TGF-β in human intestinal fibroblasts from Crohn’s disease patients and healthy controls [35] as well as the expression of TGF-β in the kidney of diabetic rats [75] and the transduction of TGF signaling in human prostate cancer cell lines [5]. Additional evidence of the inhibitory effect of T3 on TGF-β signaling has been reported in a rat model of spinal cord injury [91] and in human airway smooth muscle cells [17]. Anyway, more studies are needed to verify whether T3 may delay cellular senescence by targeting TGF-β signaling.

### Modulation of Cellular Senescence by Tocotrienols

The changes in the biomarkers of aging promoted by treatment with T3 and their marked anti-inflammatory activity are likely the consequence of the multitarget ability of these compounds. Anyway, most experimental conditions in which T3 have been tested (atherosclerosis, AD, metabolic disorders, frailty) are related to the deleterious consequences of the excessive accumulation of senescent cells. Unfortunately, whereas a multitude of studies have been addressed to investigate the mechanism of action of T3, poor attention has been given to the potential of these compounds to modulate cellular senescence [39]. In contrast to the pro-senescence activity displayed in cancer cells, there is substantial evidence that T3 may act as senescence delayers in normal cells by targeting ROS and molecular pathways related to the promotion of replicative senescence. Moreover, there is substantial evidence that T3 may target the pathways upstream of SASP production (mTOR and NF-kB), and it has been hypothesized that that they may eventually promote the selective death of senescent cells in particular experimental settings [40].

Incubation of human senescent fibroblasts at various passages with a T3 rich extract was shown to reverse the senescent morphology, to decrease the activity of SA-β-gal as well as the amount of damaged DNA and cells in G0/G1 phase, and to increase telomere length and the number of cells in the S phase [36]. Given that overexpression of the telomerase reverse transcriptase (TERT) do not revert the senescent phenotype in human fibroblasts [4], it is likely that restoration of telomerase activity is not involved in the reversal of the senescent status shown by T3. Similarly, TRF was found to reduce SA-β-gal, to ameliorate antioxidant defence mechanisms and to increase cell proliferation of myoblasts in stress-induced premature senescence [27, 28]. In another study, researchers found that T3 can prevent cellular senescence of human diploid fibroblasts by modulating a multitude of senescence-associated microRNAs (SA-miRNAs) and their target genes involved in cell cycle arrest during cellular senescence [26].

There are at least three major mechanisms that can contribute to explain the effects of T3 on senescent cells:

1. The elimination of the excess of ROS or other triggers of cellular senescence.

Senescent cells have been reported to produce an excess of ROS which can be suppressed by antioxidants with a partial reversion of the senescent phenotype [23]. Moreover, ROS are a well-established trigger of cellular senescence and may accelerate the onset of replicative senescence “in vitro” [58]. Considering that T3 have a high antioxidant potential and that they are able to modulate the Nrf2 mediated antioxidant response, the modulation of the redox status of the cells could explain both the delay of onset and the reversal of the senescent phenotype observed “in vitro” after T3 treatment. Accumulating evidence also suggests that T3 can inhibit the activity of TGF-β [5, 35, 75] that is another trigger of cellular senescence [31] and may contribute to exhaustion of stem cells in neurogenic and myogenic niche [94].

The alteration of splicing factor expression is another mechanism by which T3 could delay or reverse the senescence phenotype. It has been recently demonstrated that small molecules (such as resveratrol analogues) are able to modulate the expression of splicing factors with a subsequent rescue of multiple aspects related to cellular senescence including increased telomere length, re-entry into the cell cycle and restarted proliferation [33]. In this context, it has been reported that T3 are capable to correct aberrant splicing of IkappaB kinase complex-associated protein (IKAP) in cells derived from patients with familial dysautonomia [2] and to modulate the expression of a specific set of miRNAs in HeLa cells involved in the alternative splicing of pro-apoptotic proteins, such as the X-box binding protein 1 (XBP-1) [10]. Hence, alteration of splicing factor levels may be an additional mechanism by which T3 can reverse cellular senescence.

2) Inhibition of pathways that are responsible for SASP production.

The SASP can disrupt tissues and contribute to age-related pathologies, including cancer. The pathways that promote SASP production in senescent cells have been investigated during these last years. Previous studies showed that the SASP is regulated by a number of factors, including the transcription factor NF-κB and the MAP kinase p38 [16], and more recently it has been established that the activity of the mammalian target of rapamycin (mTOR) is upstream of these signals in senescent cells. mTOR promotes the transcription of cytokine-encoding genes via IL1A translation [32] and stabilizes their mRNAs via MK2-mediated phosphorylation [21]. In agreement with these observations, treatment with Rapamycin (an inhibitor of mTOR) is able to suppress the SASP in various model of senescent cells. In analogy with the effects of Rapamycin, a mixture of naturally occurring T3 and tocopherols extracted from palm oil/palm fruits as well as purified γ-T3 has been reported to negatively modulate mTOR pathways in breast cancer cells [57, 83]. Moreover, T3 have been reported to suppress the activation of NF-kB in several experimental models (see previous chapter focused on T3 and inflammation) [66, 79, 89].

Overall, these observations form a strong rationale to support the idea that the positive effects of T3 in age-related conditions may be the consequence of their effects on senescent cells including the suppression of the SASP and its negative impact on cells and tissues function.

3) Selection of “healthy cells” by senolysis.

Selective cell death of non-proliferating senescent cells (also termed senolysis) can be responsible for a decrease in the percentage of senescent cells and a relative increase of healthy proliferating cells, thus resembling a rejuvenating effect. This could offer an additional explanation of the effects observed “in vitro” after T3 treatment of senescent cells. Senolytic activity of T3 has not been directly tested, but there is indirect evidence that T3 may exert this activity by affecting proteostasis and promoting endoplasmic reticulum-related apoptosis in senescent cells characterized by a strong SASP response. [40]. Moreover, γ-T3 have been shown to suppress aerobic glycolysis [57] which may be a key survival factor in some senescent cells [12].

**Fig. 1:**
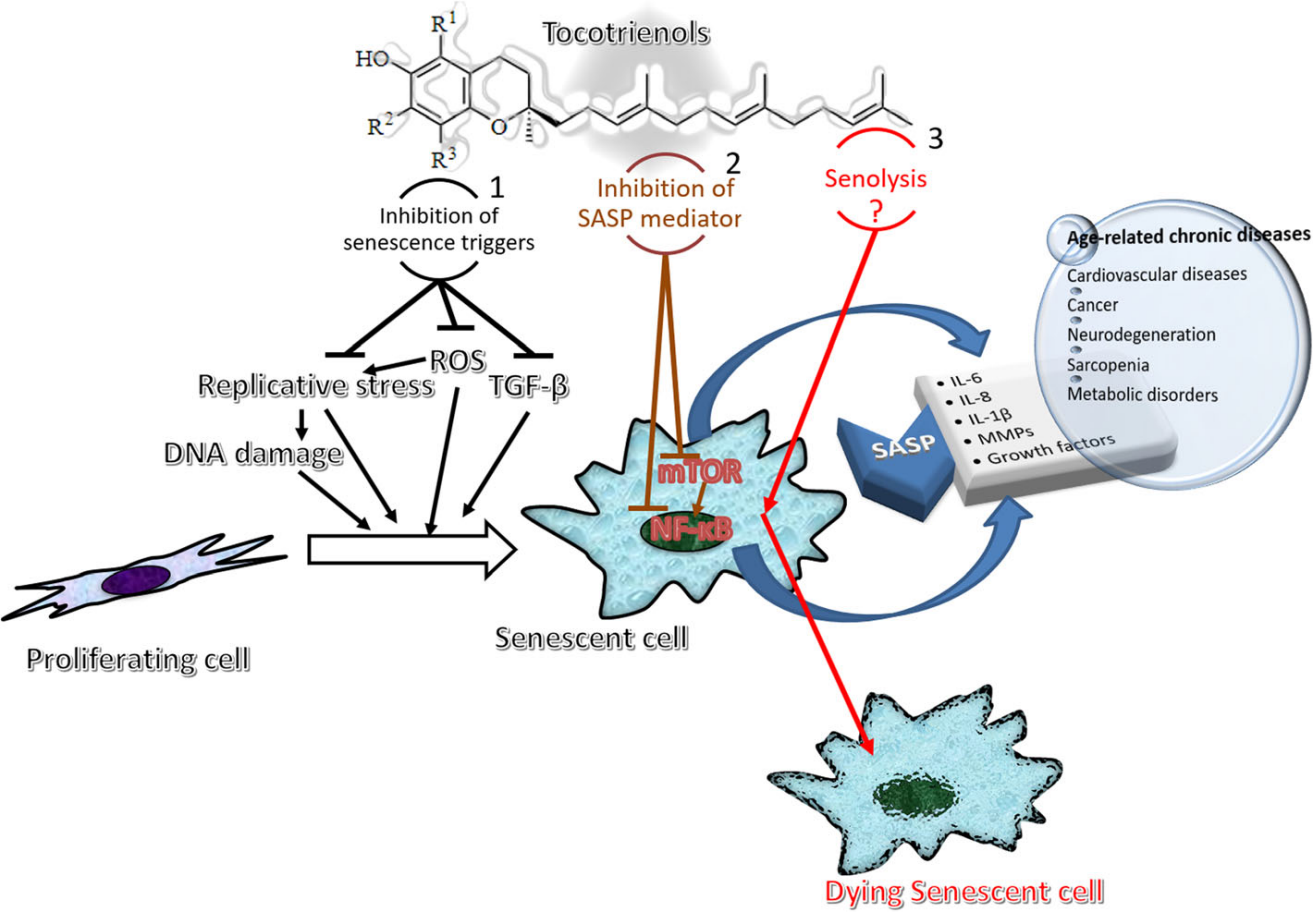
Tocotrienols have been shown to display beneficial effects in a multitude of age-related conditions related to the accumulation of senescent cells. It has been shown in vitro that tocotrienols may act as senescence delayers (1 – black arrows) by reducing the amount of ROS which, in turn, are a direct trigger of senescence or contribute to replicative and DNA-damage induced senescence. Tocotrienols may additionally inhibit the pathways that drive the production of the SASP (2 – brown arrows) by targeting mTOR and NF-kB. Finally, it has been hypothesized that tocotrienols may promote selective death of senescent cells (senolysis) (3 – red arrows) by increasing endoplasmic reticulum stress or suppressing glycolysis or by other mechanisms that still deserve to be investigated

All the above-mentioned properties of T3 that may interfere with senescent cells function and accumulation are schematically depicted in Fig. 1.

## Concluding Remarks

T3 have been reported to display a multitude of positive effects on the prevention and treatment of age-related pathologies. These include the amelioration of the lipid profile, the promotion of Nrf2 mediated cytoprotective activity and the suppression of inflammation.

All these effects may be the consequence of the ability of T3 to target multiple pathways. We here propose that these effects may be the result of a single target of T3. This target is the SASP produced by senescent cells. Indeed, T3 may act by a direct suppression of the SASP, mediated by inhibition of NF-kB and mTOR, or by removing the origin of the SASP trough senolysis. As a consequence, many age-related pathologies connected with the SASP may be attenuated or prevented by T3 treatment.

A separate case is represented by the action of T3 at the level of cancer transformation and growth. In fact, the anti-cancer effect of T3 can be exerted through at least two different mechanisms. On one hand, the inhibitory effect of T3 on SASP-related inflammation may decrease the potential risk created by a tissue microenvironment that is permissive for the development of cancer. On the other hand, T3 can directly induce cellular senescence and/or apoptosis on cancer cells, thereby inhibiting the growth and diffusion of cancer.

## Abbreviations

AGE: Advanced glycosylation end products
HDF: Human diploid fibroblasts
HMGR: 3-hydroxy-3-methyl-glutaryl-coenzyme A reductase
HUVEC: Human umbilical vascular endothelial cells
IKAP: IkappaB kinase complex-associated protein
IL-1β: Interleukin-1 beta
IL-6: Interleukin-6
LDL: Low density lipoprotein
MMPs: Matrix metalloproteinases
mTOR: Mammalian target of rapamycin
NF-kB: Nuclear factor kappa-light-chain-enhancer of activated B cells
ROS: Reactive oxygen species
SA-miRNAs: Senescence-associated microRNAs
SASP: Senescence-associated secretory phenotype
STAT3: Signal transducer and activator of transcription 3
T3: Tocotrienols
TERT: Telomerase reverse transcriptase
TGF-β: Transforming growth factor β
TNF-α: Tumor necrosis factor α
TRF: Tocotrienol-rich fraction
VCAM1: Vascular cell adhesion molecule-1
XBP-1: X-box binding protein 1
